# Efficacy and safety of atezolizumab plus bevacizumab combined with hepatic arterial infusion chemotherapy for advanced hepatocellular carcinoma

**DOI:** 10.3389/fimmu.2022.929141

**Published:** 2022-08-05

**Authors:** Yujing Xin, Fei Cao, Hongcai Yang, Xinyuan Zhang, Yi Chen, Xiaojing Cao, Xiang Zhou, Xiao Li, Jinxue Zhou

**Affiliations:** ^1^ Department of Interventional Therapy, National Cancer Center/National Clinical Research Center for Cancer/Cancer Hospital, Chinese Academy of Medical Sciences and Peking Union Medical College, Beijing, China; ^2^ Department of Interventional Radiology, The Cancer Hospital of the University of Chinese Academy of Sciences (Zhejiang Cancer Hospital), Institute of Basic Medicine and Cancer (IBMC), Chinese Academy of Sciences, Hangzhou, China; ^3^ Department of Hepatobiliary and Pancreatic Surgery, Affiliated Cancer Hospital of Zhengzhou University, Zhengzhou, China

**Keywords:** atezolizumab, bevacizumab, hepatic arterial infusion chemotherapy, FOLFOX, advanced hepatocellular carcinoma

## Abstract

**Background:**

Atezolizumab plus bevacizumab has been proved to have promising antitumor activity and tolerable safety in patients with unresectable hepatocellular carcinoma (HCC). Hepatic arterial infusion chemotherapy (HAIC) also demonstrated high response rates and favorable survival for patients with advanced HCC. This study aimed to explore the preliminary clinical efficacy and safety of atezolizumab plus bevacizumab combined with HAIC for patients with treatment-naive advanced HCC.

**Methods:**

Between October 2020 and September 2021, patients with advanced HCC who initially received atezolizumab plus bevacizumab combined with HAIC of oxaliplatin, fluorouracil, and leucovorin (FOLFOX) from three hospitals in China were reviewed for eligibility. The efficacy was evaluated by tumor response rate and survival, and the safety was evaluated by the frequency of key adverse events (AEs).

**Results:**

In total, 52 eligible patients with advanced HCC who received triple therapy were included in this study. The objective response rates (ORRs) based on mRECIST and RECIST1.1 criteria were 67.3% and 44.2%, respectively. The median progression-free survival (PFS) of patients was 10.6 months (95% CI, 8.37–13.8), and the overall survival (OS) was not reached. Extrahepatic metastasis was an independent risk factor associated with PFS. All AEs were controlled and no treatment-related deaths occurred.

**Conclusion:**

Atezolizumab plus bevacizumab combined with HAIC-FOLFOX had a significant therapeutic effect and manageable AEs in patients with advanced HCC, which may be a potential treatment option for advanced HCC.

## Introduction

Hepatocellular carcinoma (HCC) is the most common primary liver cancer and a leading cause of cancer-related death worldwide (1). China is the region with the highest incidence of HCC, accounting for nearly half of the global burden of HCC (2, 3). Patients with early-stage HCC can achieve a good prognosis by curative methods such as liver resection, local ablation, or liver transplantation. However, the prognosis of patients with advanced HCC, which accounts for the majority of HCC, remains poor due to limited treatment modalities options (4, 5). Therefore, exploring potential and efficient therapeutic strategies for advanced HCC is urgently needed to improve the prognosis of patients.

Recent advances in systemic therapies including molecular targeting agents (MTAs) and immune checkpoint inhibitors (ICIs) have modified the treatment landscape of advanced HCC (6–8). Traditionally, sorafenib or lenvatinib as first-line MTAs and cabozantinib, regorafenib, or ramucirumab as second-line MTAs can modestly prolong the survival of patients with advanced HCC (9). ICIs including nivolumab or pembrolizumab have also shown promising antitumor effects and safety for unresectable HCC (u HCC) in previous clinical trials (10, 11). However, the survival benefit of patients with advanced HCC receiving MTA or ICI monotherapy remains unsatisfactory due to the low tumor response rate and poor survival. Recently, immune-based combination therapies such as dual ICIs (12, 13), MTAs combined with ICIs (14–18), and ICIs combined with local therapy (19) have shown stronger antitumor activity and superior survival outcome than systemic monotherapy. Most notably, atezolizumab plus bevacizumab (Atezo + Bev) demonstrated statistically significant and clinically meaningful survival benefits versus sorafenib as a first-line option in patients with treatment-naive u HCC in the IMbrave150 trial (15) and became the first first-line combination therapy for the treatment of u HCC. However, the updated IMbrave150 trial found that Atezo + Bev has limited benefit in the subgroup of patients with advanced HCC with an objective response rate (ORR) of only 27% and median progression-free survival (PFS) of only 6.5 months (16), which implies that Atezo + Bev regimen may not yet achieve the optimal antitumor response in high-risk patients with advanced HCC.

Hepatic arterial infusion chemotherapy (HAIC), as locoregional interventional therapy, can enhance tumor response rate and reduce systemic toxicity by increasing local chemotherapeutic drug concentrations within tumor tissue (20–22) and has been recommended as one of the first-line options for patients with advanced HCC in the Asian region (23). Recent two high-quality HAIC Phase III trials from China demonstrated that HAIC of fluorouracil, leucovorin, and oxaliplatin (HAIC-FOLFOX) alone or combined with sorafenib had a remarkably higher ORR and superior survival than sorafenib alone in treating advanced HCC with macrovascular invasion or high tumor burden (22, 24). In addition, a few studies have reported that the combined therapy of HAIC, MTAs, and ICIs had potential therapeutic effect and tolerable safety profiles in advanced HCC (25–27). These findings suggest that HAIC may have a synergistic and positive effect in combination with MTAs or ICIs.

Although the Atezo + Bev regimen has achieved significant survival benefits in unresectable HCC, its therapeutic response in advanced HCC remains poor. HAIC can increase the anti-tumor response of Atezo + Bev by effectively reducing intrahepatic tumor burden and stimulating the exposure of tumor immune antigens (28). Moreover, bevacizumab can overcome the resistance of chemotherapy drugs through the normalization of tumor neovasculature (29). This potential synergistic effect of HAIC combined with MTAs and ICIs may further enhance the anti-cancer activity and prolong the response duration to improve the prognosis of patients (30). Therefore, we point out that Atezo + Bev combined with HAIC-FOLFOX may be a promising and efficient treatment option for patients with advanced HCC, which may provide a reference for the multidisciplinary precision treatment of HCC. Up to now, there is no study on Atezo + Bev combined with HAIC-FOLFOX in the treatment of advanced HCC. Hence, this multicenter retrospective study was performed to evaluate the safety and preliminary antitumor efficacy of Atezo + Bev combined with HAIC-FOLFOX for patients with advanced HCC.

## Methods

### Study population

Between October 2020 and September 2021, patients diagnosed with advanced HCC who had not received prior treatment from three hospitals in China (the National Cancer Center, Affiliated Cancer Hospital of Zhengzhou University, and Zhejiang Cancer Hospital) were included in this study. The ethics committee approved the ethics of this retrospective study, and all participants signed informed consent for treatment. The criteria for eligible patients were as follows: ≥18 years old, advanced HCC diagnosed as Barcelona Clinic Liver Cancer (BCLC) C stage by clinical guidelines (31), liver function was rated as Child-Pugh A class, Eastern Cooperative Oncology Group Performance Status (ECOG PS) score of 0–1, at least one measurable target lesion that can be assessed by Response Evaluation Criteria in Solid Tumors, version 1.1 (RECIST 1.1) and modified RECIST (mRECIST) (32, 33), and appropriate organ and hematologic function. The exclusion criteria included the following: the patient had previously received antitumor treatment, such as MTAs, ICIs, local interventional therapy, symptomatic brain metastasis, other malignant tumors, active autoimmune disease, incomplete medical information, and loss of follow-up.

### Treatment procedure

Eligible patients who received Atezo + Bev combined with HAIC-FOLFOX participated in this study. Specifically, patients received atezolizumab (1,200 mg) plus bevacizumab (15 mg/kg) intravenously once every 3 weeks followed by HAIC-FOLFOX. The procedure of the HAIC-FOLFOX was as follows: First, the femoral artery was percutaneously punctured using Seldinger’s technique. Then, the 5-Fr catheter was inserted into the celiac trunk or superior mesenteric artery for arteriography, and a 2.7-Fr microcatheter was super selectively placed into the feeding arteries of the tumor and the tumor thrombus. When blood flows into the gastroduodenal artery was confirmed by micro-catheter angiography, the route was embolized with a coil or micro-coil to prevent reflux of chemotherapeutic drugs to the stomach and duodenum. The peripheral end of the microcatheter was locked with a heparin lock (10 ml, 10,000 units, 1:1,000 dilution) to prevent clotting of the catheter. The peripheral part of the catheter that exposing to the outside of the body was covered with medical sterile gauze and fastened on the skin of the thigh using medical rubberized fabric and bandage. Medication was started within 2 days after catheter insertion. The following chemotherapeutic agents were sequentially infused into the hepatic artery by connecting an arterial pump: oxaliplatin at 85 mg/m^2^ for 2–4 h, leucovorin at 400 mg/m^2^ for 2 h, fluorouracil at 400 mg/m^2^ for 1 h, and another fluorouracil at 2,400 mg/m^2^ for more than 46 h (20, 34, 35). After treatment, catheters were removed from the patient. Catheter insertion was repeatedly performed before every cycle of treatment. To ensure safety and completion of treatment, this study extended the treatment interval of HAIC to 4–6 weeks, which can reduce the number of hospitalizations and medical costs, with the efficacy of treatment unaffected.

### Dose adjustment in treatment

Disease progression and intolerable toxicity may lead to the interruption of treatment or dose adjustment. Atezolizumab or bevacizumab may be transiently or permanently discontinued in case of grade 3–4 adverse events (AEs), and dose adjustment of HAIC-FOLFOX was based on previous clinical trials (20, 22). Specifically, the dose of 5-fluorouracil would be reduced to 300 mg/m^2^ bolus and 1,800 mg/m^2^ per cycle when grade 3 or 4 AEs occur, such as grade 3–4 diarrhea, skin toxicity, or stomatitis, and any other grade 3 major organ treatment-related toxicity. The dose of oxaliplatin would be reduced to 65 mg/m^2^ per cycle when grade 3 or 4 AEs occur, for example, grade 3 or 4 thrombocytopenia or neutropenia, or treatment-related abdominal pain, and any other grade 3 major organ treatment-related toxicity. In addition, HAIC was delayed if neutrophil count ≤1,200 cells/μl, platelet count ≤60,000 platelets/μl, a total bilirubin level ≥30 mmol/L, an albumin level ≤3.0 mg/dL, or serum creatinine level reaches 1.5 times the upper limit of normal.

### Data collection and follow-up

The clinical baseline characteristics and follow-up data of eligible patients were collected and analyzed through medical records, including sex, age, cirrhosis, ECOG PS score, etiology, maximum tumor size, tumor number, α-fetoprotein (AFP) level, alanine aminotransferase (ALT), albumin-bilirubin (ALBI) grade, albumin (ALB), aspartate aminotransferase (AST), total bilirubin(TBIL), presence or absence of macrovascular invasion and/or extrahepatic metastasis, and classification of portal vein tumor thrombus (PVTT). Liver cirrhosis was diagnosed through non-invasive tests such as medical imaging, liver function indicators, and etiology recommended by the liver cirrhosis guidelines of EASL and Chinese Society of Hepatology (36, 37). Imaging was evaluated and reviewed by experienced radiologists.

The last follow-up of this study was 31 March 2022. Tumor response was assessed by contrast-enhanced MR or CT every 4 to 8 weeks, and the patient’s follow-up data were recorded every 2 months until the disease progression or death.

### Outcomes and assessments

The primary endpoints were ORR and PFS. Tumor responses were classified as progressive disease (PD), stable disease (SD), partial response (PR), and complete response (CR) based on mRECIST and RECIST version 1.1. The ORR was referred to as the sum of PR and CR. The disease control rate (DCR) referred to the sum of PR, SD, and CR. PFS referred to the time from the beginning of the initial combination therapy to the progression of the disease or death. The secondary endpoints were incidence of AEs and overall survival (OS). OS referred to the time interval from initial combination therapy to death from any cause. AEs were evaluated by the National Cancer Institute Common Terminology Criteria for Adverse Events version 5.0.

### Statistical analysis

Tumor response, survival, and AEs were assessed and analyzed in patients who received at least two cycles of triple therapy. Baseline characteristics and response rates are expressed in terms of frequencies and percentages, and variables are indicated as either the mean (range) or median (standard deviation). The Kaplan–Meier method was used to estimate PFS, and univariate and multivariate regression analyses were used to analyze the prognostic factors of PFS. All analyses were performed using SPSS version 25.0.

## Results

### Patient and tumor characteristics

**Figure 1:**
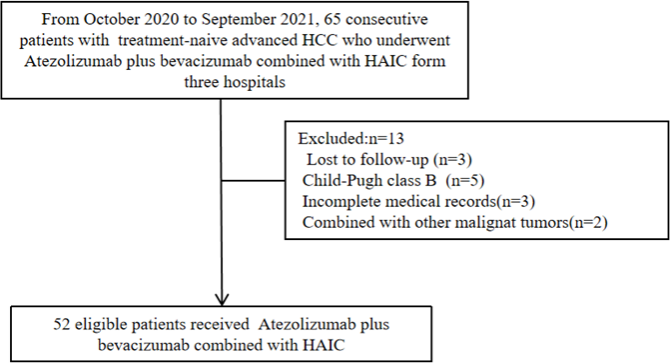
Patient flow chart.

As of the end of follow-up, a total of 52 eligible patients with advanced HCC receiving Atezo + Bev combined with HAIC-FOLFOX were enrolled in this study (**Figure 1**). The baseline characteristics are summarized in **Table 1**. The patients in this study had BCLC stage C HCC with a high tumor burden, and the average size of the maximum tumor was 10.2 cm. Thirty-seven (71.2%) patients had portal vein tumor thrombus, and 26 (50.0%) patients had extrahepatic metastasis. Most of the patients were men and infected with the hepatitis B virus.

**Table 1:** Patient demographics and clinical characteristics.

Characteristics	Number	Percentage (%)
Sex		
Male	46	88.5%
Female	6	11.5%
Age (years) ± SD	55.9 ± 11.1	
<55	20	38.5%
≥55	32	61.5%
ECOG-PS		
0	49	94.2%
1	3	5.8%
Etiology		
Hepatitis B	47	90.4%
Others	5	9.6%
Cirrhosis		
Yes	24	46.2%
No	28	53.8%
ALBI grade		
1	43	82.7%
2	9	17.3%
ALB(g/l)		
<40	14	26.9%
≧̸40	38	73.1%
TBIL (μmol/l)		
≦̸20	36	69.2%
>20	16	30.8%
AFP (ng/ml)		
<400	22	42.3%
≥400	30	57.7%
AST (U/L)		
≤40	14	26.9%
>40	38	73.1%
ALT (U/L)		
≤50	35	67.3%
>50	17	32.7%
Tumor size(mean ± SD, cm)	10.2 ± 3.0	
<10	22	42.3%
≥10	30	57.7%
Tumor number		
Single	4	7.7%
Multiple	48	92.3%
Tumor thrombus		
Absent	15	28.9%
Vp1-2	19	36.5%
Vp3	8	15.4%
Vp4	10	19.2%
Extrahepatic metastasis		
Yes	26	50.0%
No	26	50.0%

AFP, α-fetoprotein; ALBI, albumin-bilirubin; ALT, alanine aminotransferase; AST, aspartate aminotransferase; TBIL, total bilirubin; ECOG PS, Eastern Cooperative Oncology Group Performance Status; Vp1, third branch portal vein invasion; Vp2, second branch portal vein invasion; Vp3, first branch portal vein invasion; Vp4, main portal vein invasion.

### Treatment efficacy

**Figure 2:**
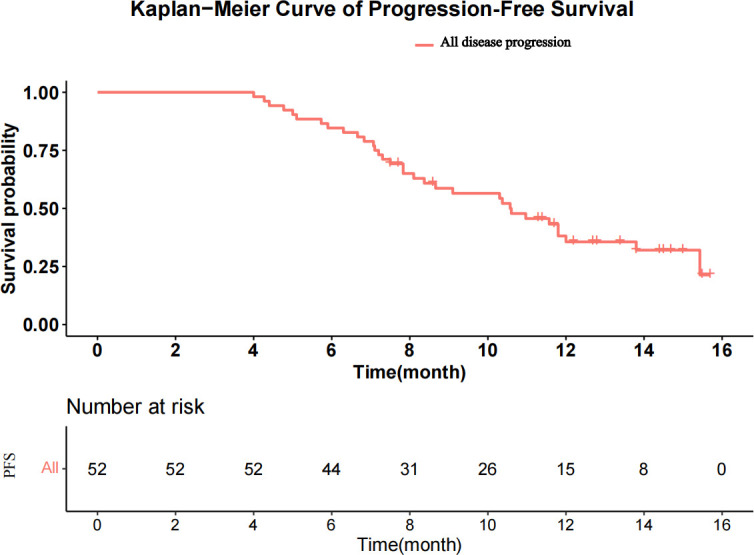
Kaplan–Meier curves for progression-free survival.

The median follow-up was 15.6 months (range, 8.5–17.8 months). A total of 213 HAIC cycles were performed during the study, with a median of four cycles (range, two to eight cycles). The median duration of Atezo + Bev was 8.7 months (range, 4.0–15.7 months). At the time of analysis, 33 patients had disease progression, and 10 patients had died in the study. The median PFS was 10.6 months [95% confidence interval (CI), 8.37–13.8; **Figure 2**], and the median OS was not reached. The 3-, 6-, and 12-m PFS rates were 100.0%, 84.6%, and 35.6%, respectively. The 3-, 6-, and 12-month OS rates were 100.0%, 96.2%, and 86.5%, respectively.

**Figure 3:**
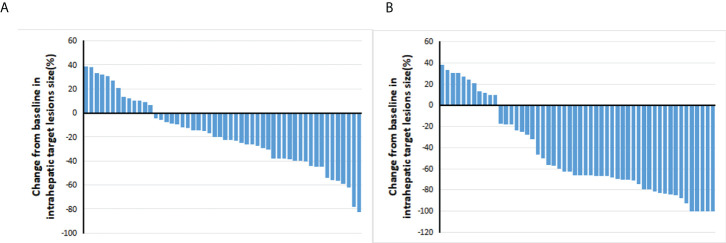
Best percentage changes from baseline in the size of the intrahepatic target lesions of patients receiving atezolizumab plus bevacizumab combined with HAIC. **(A)** Assessed with RECIST1.1 in patients with image measurements before and after treatment. **(B)** Assessed with mRECIST in patients with image measurements before and after treatment.

**Figure 4:**
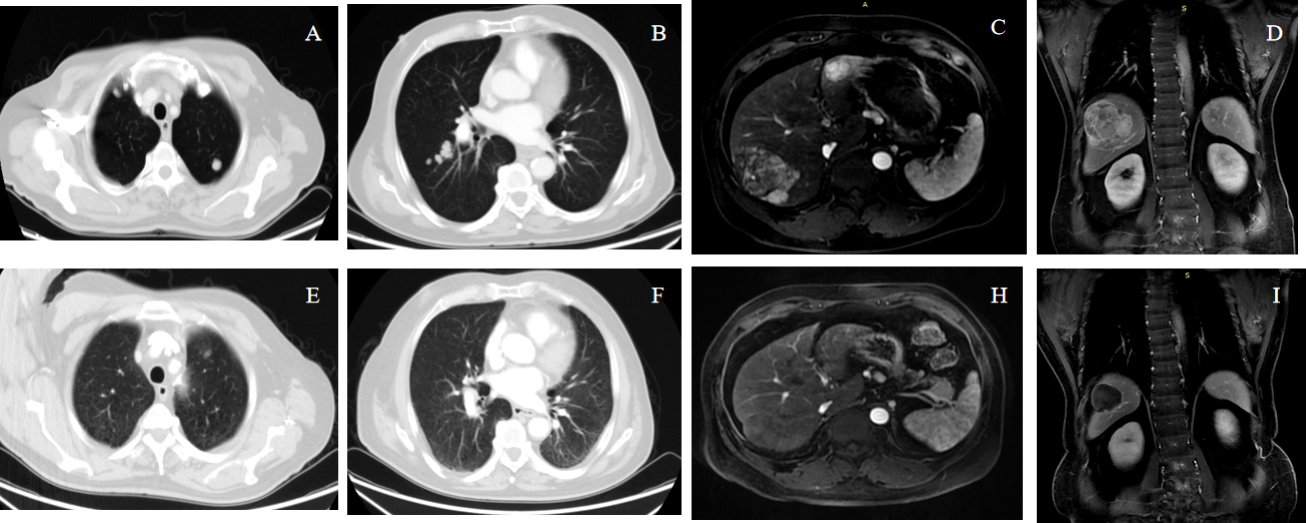
A 68-year-old male patient with advanced HCC with lung metastases achieved complete response (mRECIST) and partial response (RECIST1.1) after receiving atezolizumab plus bevacizumab combined with HAIC-FOLFOX. Panels **(A–D)** show the pre-treatment images, and Panels **(E–I)** show the post-treatment.

The tumor responses are shown in **Table 2**. The ORR based on RECIST 1.1 and mRECIST criteria were 44.2% and 67.3%, respectively. Five patients (9.6%) achieved CR according to the mRECIST criteria, and the DCR was 76.9%. The change in the intrahepatic target lesion size of patients is shown in **Figures 3A,B**. A patient was evaluated as CR based on the mRECIST criteria after treatment with HAIC combined with Atezo + Bev, as shown in **Figures 4A-I**.

**Table 2:** Radiological response according to mRECIST and RECIST1.1.

	mRECISTN (%)	RECIST1.1N (%)
Best response		
CR	5 (9.6%)	0 (0%)
PR	30 (57.7%)	23 (44.2%)
SD	5 (9.6%)	17 (32.7%)
PD	12 (23.1%)	12 (23.1%)
ORR (CR+PR)	35 (67.3%)	23 (44.2%)
DCR (CR+PR+SD)	40 (76.9%)	40 (76.9%)

CR, complete response; PD, progressive disease; PR, partial response; SD, stable disease; DCR, disease control rate; ORR, objective response rate; mRECIST, modified Response Evaluation Criteria in Solid Tumors; RECIST, Response Evaluation Criteria in Solid Tumors.

### Safety and tolerability

Treatment-related deaths did not occur in this study, and the frequency of the key AEs of all grades was 92.3% (48 AEs in 52 patients). The treatment-related AEs (TRAEs) that occurred in ≥10% of patients are shown in **Table 3**. The most common grade 1–2 AEs included nausea (42.3%), fatigue (38.5%), and elevated AST (34.6%). The most common grade 3–4 AEs were hypertension (7.7%), elevated AST (5.8%), and thrombocytopenia (3.8%). Grade 1–2 hypothyroidism (15.3%) was the most common potentially immune-related TRAE. Any grade liver dysfunction in most patients, such as elevated AST, elevated ALT, and hyperbilirubinemia, was mainly mild to moderate and returned to normal after treatment. Furthermore, specific abdominal pain associated with the HAIC of oxaliplatin occurred in 20 (38.4%) patients in the study. This pain could be acute and severe but was quickly relieved by slowing or stopping the infusion of oxaliplatin, after which the infusion would continue. On the other hand, only two patients (3.8%) discontinued Atezo + Bev treatment for grade 4 AEs, and five patients (9.6%) had dose reductions of HAIC for grade 3–4 adverse reactions, but no patients discontinued HAIC treatment.

**Table 3:** Treatment-related adverse events occurring at ≥10% incidence for all grades.

AEs, n (%)	Any Grade	Grades 1–2	Grade 3	Grade 4
Nausea	23 (44.2%)	22 (42.3%)	1 (1.9%)	0 (0.0%)
Fatigue	22 (42.3%)	20 (38.5%)	2 (3.8%)	0 (0.0%)
Elevated AST	21 (40.4%)	18 (34.6%)	3 (5.8%)	1 (1.9%)
Abdominal pain	20 (38.4%)	18 (34.6%)	2 (3.8%)	0 (0.0%)
Elevated ALT	18 (34.6%)	16 (30.8%)	2 (3.8%)	1 (1.9%)
Hypertension	17 (32.7%)	13 (25.0%)	4 (7.7%)	2 (3.8%)
Vomiting	16 (30.7%)	14 (26.9%)	2 (3.8%)	0 (0.0%)
Proteinuria	15 (28.8%)	13 (25.0%)	2 (3.8%)	1 (1.9%)
Thrombocytopenia	14 (26.9%)	12 (23.1%)	2 (3.8%)	1 (1.9%)
Hyperbilirubinemia	11 (21.2%)	11 (21.2)	0 (0.0%)	0 (0.0%)
Leukopenia	10 (19.2%)	8 (15.4%)	2 (3.8%)	0 (0.0%)
Decreased appetite	9 (17.3%)	8 (15.4%)	1 (1.9%)	0 (0.0%)
Hypothyroidism	8 (15.3%)	8 (15.3%)	0 (0.0%)	0 (0.0%)
Diarrhea	7 (13.4%)	6 (11.5%)	1 (1.9%)	0 (0.0%)
Pyrexia	7 (13.4%)	7 (13.4%)	0 (0.0%)	0 (0.0%)
Neutropenia	7 (13.4%)	6 (11.5%)	1 (1.9%)	0 (0.0%)
Weight decrease	5 (10.4%)	5 (10.4%)	0 (0.0%)	0 (0.0%)

ALT, alanine aminotransferase; AST, aspartate aminotransferase.

### Prognostic factor analysis

**Figure 5:**
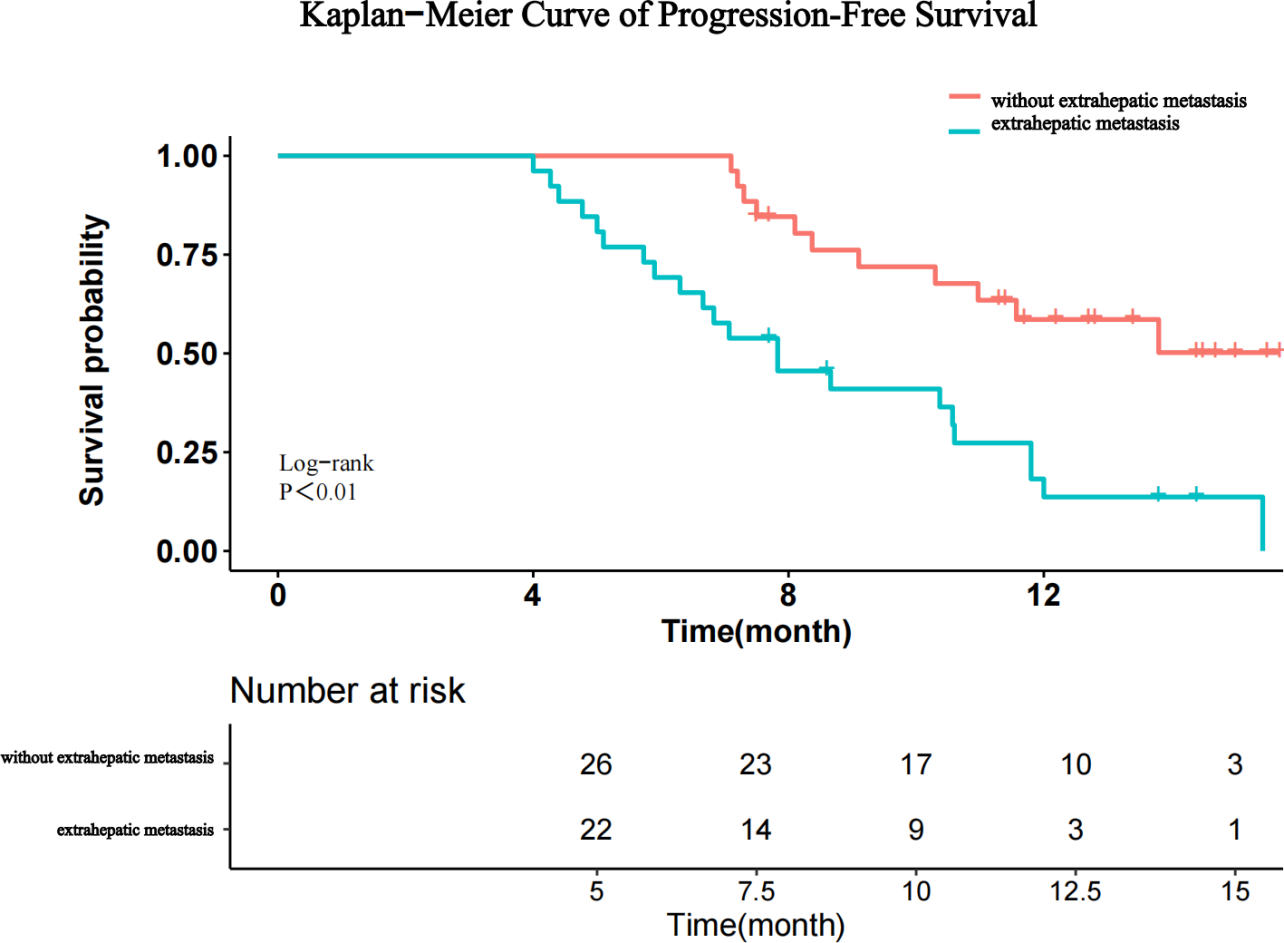
Kaplan–Meier curves for progression-free survival after stratification by the absence or presence of extrahepatic metastasis.

The prognostic factors for PFS are shown in **Table 4**. Univariate analysis showed that ALB, AFP level, and extrahepatic metastasis were related factors for PFS, and multivariate analysis showed that extrahepatic metastasis was an independent risk factor for PFS. Patients with advanced HCC without extrahepatic metastases had longer PFS than those with extrahepatic metastases (**Figure 5**).

**Table 4:** Univariate and multivariate analyses of prognostic factors affecting PFS in advanced HCC.

Variables	Univariate Cox Analysis			Multivariate Cox Analysis		
	HR	95% CI	*P*-value	HR	95% CI	*P*-value
Sex (male *vs*. female)	0.97	0.30–3.20	0.962			
Age (<55 *vs*. ≥55)	1.86	0.88–3.93	0.104			
ECOG-PS (0 *vs*. 1)	3.13	0.71–13.88	0.133			
Etiology (hepatitis B *vs*. others)	0.59	0.18–1.94	0.380			
Cirrhosis (yes *vs*. no)	1.04	0.52–2.06	0.923			
ALBI (1 *vs*. 2)	1.43	0.64–3.21	0.380			
ALB (<40 *vs*. ≥40)	2.40	0.99–5.8	0.053	1.89	0.77–4.63	0.163
TBIL (<20 *vs*. ≥20)	0.82	0.39–1.75	0.614			
AFP (≥400 ng/ml *vs*. <400 ng/ml)	1.99	0.96–4.14	0.064	1.52	0.71–3.20	0.289
ALT (≥50 U/L *vs*. <50 U/L)	0.86	0.41–1.82	0.697			
AST (≥40 U/L *vs*. <40 U/L)	0.58	0.28–1.21	0.148			
Tumor size (≥10 cm *vs*. <10 cm)	1.71	0.83–3.51	0.142			
Tumor node (multiple *vs*. single)	2.28	0.54–9.57	0.260			
Macroscopic vascular invasion (yes *vs*. no)	0.94	0.44–2.03	0.879			
Extrahepatic metastasis (yes *vs*. no)	3.39	1.64–7.03	0.001	2.80	1.32–5.92	0.007

AFP, α-fetoprotein; ALBI, albumin-bilirubin; ALT, alanine aminotransferase; AST, aspartate aminotransferase; TBIL, total bilirubin; ECOG PS, Eastern Cooperative Oncology Group Performance Status.

## Discussion

The preliminary results of this multicenter retrospective study demonstrated that the triple therapy of Atezo + Bev combined with HAIC has high ORR (67.3%) and a median PFS (10.6 m) in advanced HCC with acceptable safety profiles, indicating that novel triple therapy may bring satisfactory efficacy and favorable survival to patients with treatment-naive advanced HCC.

This study first evaluated the potential therapeutic efficacy of Atezo + Bev combined with HAIC in advanced HCC. In this study, most patients had PVTT (71.2%), extrahepatic metastasis (50.0%), or high tumor burden (57.7%), and the survival benefit of these patients was considered to be extremely poor. However, our study found that Atezo + Bev combined with HAIC can significantly improve the prognosis of patients with advanced HCC with high response rates and favorable survival. Both ORR and PFS of triple therapy were superior to those reported in previous studies with Atezo + Bev and other first-line TKIs therapy in advanced HCC, with an ORR of 2% to 27.3% and a PFS of 3.7–7.3 months (16, 38, 39). Notably, we observed that five patients (9.6%) achieved CR and seven patients (13.4%) were downstaged from advanced HCC to intermediate HCC, which suggested that these beneficiaries may receive curative treatment earlier and achieve longer disease-free survival. Multivariate analysis in this study showed that extrahepatic metastasis is an independent risk factor for PFS, which is consistent with the previous studies (40). Therefore, we should focus on exploring the immunosuppressive microenvironment and its drug resistance mechanism of HCC with extrahepatic metastasis in the future.

The synergistic effect of combination therapy with different mechanisms has been confirmed in HCC treatment (41, 42). The high response rate and improved survival found in our study may be caused by the synergistic effect of atezolizumab, bevacizumab, and HAIC-FOLFOX. First, not only HAIC can reduce tumor burden by maintaining high concentrations of chemotherapy in the tumor but also the immunogenic cell death induced by chemotherapy can enhance the antitumor effect of ICIs (43, 44). Second, bevacizumab can enhance the antitumor activity of atezolizumab by inhibiting immunosuppression and promoting the infiltration of immune T cells in the tumor microenvironment (45–47). On the other hand, atezolizumab combined with bevacizumab may disrupt the hypoxic microenvironment within tumors by normalizing tumor vessels and overcoming resistance to FOLFOX agents (22). Therefore, triple therapy with atezolizumab, bevacizumab, and HAIC can rapidly reduce tumor burden, prolong the response time of systemic therapy, and further prolong the long-term survival of patients. In addition, our study found that the ORR in patients with PVTT was 46.2%, and the ORR of patients with Vp1-3 type was higher than that of patients with Vp4 type. This suggests that we should apply combination therapy in the early stage before the portal vein tumor thrombus invades the main trunk, which can effectively inhibit the growth of tumor thrombus, reduce portal pressure, and protect liver function.

The AEs observed in this study were relatively common with triple therapy, and there were no deaths related to AEs. Although grade 3–4 hypertension and elevated AST caused by combined therapy were common, they could be controlled by adjusting drug dose and discontinuing treatment. In our studies, the TRAEs related to Atezo + Bev, including hypothyroidism, proteinuria, and hypertension, were consistent with those reported in the previous IMbrave150 (15, 16). HAIC-related TRAEs, such as liver dysfunction, nausea, and specific abdominal pain, were also consistent with previously reported findings (21, 35, 48). We did not observe other potential synthetic toxicity events in this study, which proved that triple therapy is clinically feasible and safe.

Although our study reported preliminary clinical results of Atezo + Bev combined with HAIC for advanced HCC and thus provides clinical evidence for future prospective trials, there were some limitations. First, the present study was a retrospective study conducted in China, which was performed in three institutions, with limited sample size and potential bias. Many patients withdrew from the study due to high medical costs, fear of complications, and the prevalence of COVID-19. Therefore, this study needs to be expanded to larger populations in Western countries and other regions. Second, the dosing intensity and adverse reactions of drugs may be underestimated due to retrospective analysis, so the results of this study need to be validated in a prospective multicenter randomized clinical trial in the future. Third, the OS of patients receiving triple therapy has not yet been reached due to the short follow-up time, but the short-term efficacy including tumor response rate and PFS was fully evaluated in this study and was not affected by subsequent treatment, which more accurately represents the clinical efficacy of combination therapy than OS. In the future, we will continue to follow up and obtain long-term survival data. Finally, this study mainly focused on HBV-related HCC, whether this combination therapy can be applied to patients with HCC with other etiologies needs further research.

In summary, Atezo + Bev combined with HAIC-FOLFOX had a significant therapeutic effect and manageable AEs in patients with treatment-naive advanced HCC. Thus, this triple therapy of atezolizumab, bevacizumab, and HAIC may become an alternative and promising treatment for HCC.

## Data availability statement

The original contributions presented in the study are included in the article/Supplementary Material. Further inquiries can be directed to the corresponding author/s.

## Ethics statement

This study was reviewed and approved by National Cancer Center/National Clinical Research Center for Cancer/Cancer Hospital, Chinese Academy of Medical Sciences and Peking Union Medical College, Beijing, 100021, China. The patients/participants provided their written informed consent to participate in this study. Written informed consent was obtained from the individual(s) for the publication of any potentially identifiable images or data included in this article.

## Author contributions

The authors of this manuscript declare no relationships with any companies, whose products or services may be related to the subject matter of the article. (I) Conception and design: YX, XZ. (II) Administrative support: YX, YH, FC. (III) Provision of study materials or patients: YH, FC. (IV) Collection and assembly of data: XYZ, YC. (V) Data analysis and interpretation: YX, XC, JZ, XL. (VI) Manuscript writing: All authors (VII) Revising the manuscript: FC, YX. All authors contributed to the article and approved the submitted version.

## Conflict of interest

The authors declare that the research was conducted in the absence of any commercial or financial relationships that could be construed as a potential conflict of interest.

## Publisher’s note

All claims expressed in this article are solely those of the authors and do not necessarily represent those of their affiliated organizations, or those of the publisher, the editors and the reviewers. Any product that may be evaluated in this article, or claim that may be made by its manufacturer, is not guaranteed or endorsed by the publisher.
